# The Association between Trajectories of Anthropometric Variables and Risk of Diabetes among Prediabetic Chinese

**DOI:** 10.3390/nu13124356

**Published:** 2021-12-03

**Authors:** Fang Li, Lizhang Chen

**Affiliations:** 1 Department of Epidemiology and Health Statistics, Xiangya School of Public Health, Central South University, Changsha 410078, China; lifang_csu@csu.edu.cn; 2 Hunan Provincial Key Laboratory of Clinical Epidemiology, Changsha 410078, China

**Keywords:** prediabetes, diabetes, trajectories of body mass index, trajectories of mid-upper arm circumference, growth mixture modeling, cohort study

## Abstract

In order to explore the association between trajectories of body mass index (BMI) and mid-upper arm circumference (MUAC) and diabetes and to assess the effectiveness of the models to predict diabetes among Chinese prediabetic people, we conducted this study. Using a national longitudinal study, 1529 cases were involved for analyzing the association between diabetes and BMI trajectories or MUAC trajectories. Growth mixture modeling was conducted among the prediabetic Chinese population to explore the trajectories of BMI and MUAC, and logistic regression was applied to evaluate the association between these trajectories and the risk of diabetes. The receiver operating characteristic curve (ROC) and the area under the curve (AUC) were applied to assess the feasibility of prediction. BMI and MUAC were categorized into 4-class trajectories, respectively. Statistically significant associations were observed between diabetes in certain BMI and MUAC trajectories. The AUC for trajectories of BMI and MUAC to predict diabetes was 0.752 (95% CI: 0.690–0.814). A simple cross-validation using logistic regression indicated an acceptable efficiency of the prediction. Diabetes prevention programs should emphasize the significance of body weight control and maintaining skeletal muscle mass and resistance training should be recommended for prediabetes.

## 1. Introduction

Prediabetes is characterized by glycaemic parameters above normal but below diabetes thresholds, which includes three circumstances: impaired glucose tolerance (IGT) defined as 2-hour post-load plasma glucose (2hPG) of 7.8–11.0 mmol/L based on oral glucose tolerance test (OGTT), impaired fasting glucose (IFG) defined as fasting plasma glucose (FPG) of 5.6–6.9 mmol/L (in the absence of IGT), and hemoglobin A1c (HbA1c) levels of 5.7–6.4% [1]. Globally, the prevalence of prediabetes among adults varies widely, with an estimation of 9.0–40.0% [2]. In China, the prevalence of prediabetes is high and rapidly increasing, which reached 37.0% in 2017 [3,4].

Prediabetes is a well-acknowledged high-risk state for diabetes development [5]. Around 5–10% of people with prediabetes become diabetic annually and up to 70% of individuals with prediabetes eventually develop diabetes [5]. A 20-year Chinese diabetes prevention trial reported an even higher cumulative incidence rate of diabetes (>90%) among individuals with an IGT identified with repeated OGTT [6]. Thus, individuals with prediabetes are threatened by an increased risk of numerous complications of diabetes in the future, such as macrovascular complications (for example, cardiovascular disease) and microvascular complications (for example, complications affecting the kidney, the retina, and the nervous system) [7,8,9]. Moreover, the hyperglycemia status in the prediabetes stage impairs the kidney, nervous system, retina, and macro-vessels before the disorder progressed to diabetes [10,11,12]. These prediabetic-related diseases and disorders have burdened the family and society heavily; thus, it is crucial to identify and treat individuals with prediabetes and prevent their future development of diabetes and complications.

Abundant evidence pointed out that overweight and obesity are the risk factors of prediabetes [13,14]. Several studies also revealed the trajectories of body mass index (BMI) associated with the risk of diabetes among the general population [15,16,17]. However, the number of studies is limited with respect to focusing on the association between the trajectories of anthropometric variables (such as BMI) and risk of diabetes among individuals with prediabetes, and the highly risky population requires more attention. The only study on this issue was conducted by Huan Hu and colleagues, who studied trajectories of BMI and waist circumference (WC) among Japanese with prediabetes [18]. However, no such study was documented in China. The mid-upper arm circumference (MUAC) was a measurement of the sum of the muscle and subcutaneous fat in the upper arm [19]. Previous studies pointed out that large MUAC was associated with insulin resistance [20,21], which was a status prone to diabetes. However, the evidence was lacking with respect to the association between trajectories of mid-upper arm circumference (MUAC) and risk of diabetes among the persons with prediabetes. Considering these backgrounds, it is necessary to conduct a study to better understand the dynamic process of prediabetes to diabetes under the Chinese background, which may help to shed light on the prevention of diabetes especially among high-risk individuals in China.

Thus, we conducted this study aiming to (1) describe trajectories of anthropometric variables (BMI and MUAC) among Chinese with prediabetes, (2) to explore the association between the mentioned above trajectories and diabetes, and to (3) assess the effectiveness of models using trajectories of anthropometric variables to predict diabetes. 

## 2. Materials and Methods

### 2.1. Study Design and Population

We used the data from the China Health and Nutrition Survey (CHNS), which was a national longitudinal study that began in 1989. A multistage, random cluster process was used in CHNS every 2 to 4 years to collect data from individuals and households and their communities to understand how the various social and economic changes in China affect a wide array of nutrition and health-related outcomes [22]. Until 2015, this process involved a total of 42,829 individuals from 11,130 households and 388 communities. Further information on the CHNS is provided on the website [23]. For this study, data from CHNS of 2009, 2011, and 2015 were analyzed.

### 2.2. Ethical Approval

The study protocol of CHNS was approved by the institutional review board from the University of North Carolina at Chapel Hill and the National Institute for Nutrition and Food Safety, China Centre for Disease Control and Prevention. Written informed consent was collected from all participants. The work presented in this paper was approved by the ethical committee of Xiangya School of Public Health of Central South University (No. XYGW-2021-03) and follows the ethical principles of the Declaration of Helsinki 1964.

### 2.3. Data Collection

Biomarker data were only collected in the 2009 wave. All individuals older than seven years old (seven years old included) are required have 12 mL of blood (in three 4 mL tubes) collected on an empty stomach [24]. Fasting glucose was measured by serum using the GOD-PAP method (Randox, Crumlin, County Antrim, UK; Hitachi 7600), and HbA1c were measured by whole fresh blood using the HLC/HLC/HPLC method (Tosoh, Minato-ku, Tokyo, Japan/Bio-Rad, Hercules, CA, USA/Primus, Kansas City, MO, USA; HLC-723 G7/D10/PDQ A1c) [25]. All samples were analyzed in a national central lab in Beijing (medical laboratory accreditation certificate ISO 15189:2007) with strict quality control [26]. 

The information of outcome was collected by participants’ self-report status on diabetes in 2011 or 2015. The individuals would be reckoned as new onset of diabetes if they answered yes in the question “Has the doctor ever given you the diagnosis of diabetes?” in the wave of 2011 or 2015. An individual who answered “I do not know” would be eliminated in the final analysis. 

The anthropometric variables, including height, weight, MUAC, and triceps skinfold (TSF), were measured by physical examinations in three waves (2009, 2011, and 2015). Height was measured by using a calibrated scale at head level with the participant standing barefoot and documented to the nearest 0.1 cm. Weight was measured using a balance scale and documented to the nearest 0.1 kg. The MUAC was measured using a flexible non-stretch tape laid at the midpoint between the acromion and olecranon to the nearest 0.1 cm. The TSF was measured by skinfold calipers at a vertical pinch at the mid-point between the acromial and the radial, with arm relaxing and palm forwards. The body mass index (BMI) was calculated using height and weight and then categorized into underweight (<18.5 kg/m^2^), normal (18.5–23.9 kg/m^2^), overweight (24.0–27.9 kg/m^2^), and obese (≥28 kg/m^2^) [27]. 

Covariates were collected by interviews in three waves (2009, 2011, and 2015). These included social-demographic variables (such as age, location, ethnicity, sex, and highest educational level), lifestyle factors (smoking or not at the baseline, drinking or not at the baseline, and drinking tea or not at the baseline), energy intake at baseline, carbohydrate intake at baseline, and activity level at baseline. 

Dietary information was collected using the 24-h individual recall method on three consecutive days by trained field interviewers. With the assistance of food models and pictures, trained field interviewers asked individuals to recall food consumption and then recorded information such as the types of food, amounts of food, type of meal, and place of consumption of all food items within 24 h. The averages of energy and carbohydrate intake values were calculated by linking the dietary data linked with a nutrient databank for the new version of Chinese food composition tables [28]. 

Information of activity level was collected by self-report information. Participants answered questions on physical activity involved in work and questions related to energy-expenditure, such as “How much time did you spent on the light/middle/heavy physical activities?” [29] Examples of different activity levels were given to interviewees in order to help them better quantify the daily physical activities [30]. Then, these data were categorized into four levels: light, middle, heavy, and no working ability.

### 2.4. Statistical Analysis 

For statistical description, if continuous variables were normally distributed, they were presented as mean and standard deviation; if not, they were presented using medians and interquartile ranges. Categorical variables were descripted by numbers and proportions. Continuous variables were compared using one-way ANOVA or Kruskal–Wallis tests and categorical variables chi-square tests or Fisher’s exact tests, respectively [31]. 

Using Mplus (Version 7.4, developed by Muthén & Muthén, Los Angeles, CA, USA) [32], the growth mixture model (GMM) approach was used to model BMI and MUAC trajectories over time and to identify distinct subgroups following similar patterns. GMM is one of the most flexible clustering analyses in recent years and was applied to group individuals into an optimal number of classes or subgroups of anthropometric variables levels, such as BMI [33,34]. The model fit was assessed by the adjusted Bayesian Information Criterion (aBIC) and entropy: A small number of aBIC indicated a better-fitting model [35] and a large value of entropy represented less likelihood of misclassification [35,36]. The adjusted Lo–Mendell–Rubin likelihood ratio test (aLMR-test) and a bootstrapped likelihood ratio test (BLRT) were used to compare the n-class model versus the n-1 class models [37]. The significant *p*-value (*p* < 0.05) suggested that the n-class model was better than the n-1-class model. A scree plot was applied to comprehensively examine the fitness of models when the mentioned above model-fitness indices contradict each other [38]. 

The analyses of associations between BMI or MUAC trajectories and diabetes were conducted by using logistic regression. Odds ratios (OR) and correspondence 95% confidence intervals (CI) were applied to describe the associations. The BMI or MUAC trajectories and covariates were used as potential predictors for diabetic risk among the study population. The simple two-fold cross-validation was applied using logistic regression, with 70% of participants (n_1_ = 1070) comprising the training set and the rest (n_2_ = 459) comprising the validating set. The best cut-off point discovered from the training set was applied to the validating set to test the efficiency of prediction. The receiver operating characteristic curve (ROC) and the area under the curve (AUC) were also applied to assess the feasibility of prediction. 

Missing values of continuous variables were estimated by the EM algorithm based on the maximum likelihood while the ones of categorical variables were processed by the multiple imputation method [39,40,41]. Similar logistic regression was conducted among individuals without missing values to assess the effect of the missing values.

Statistical analyses were conducted by IBM SPSS (Version 25.0) and R (Version 4.0.4). The significant level was *p* < 0.05 unless otherwise specifically mentioned. 

## 3. Results

For this study, we extracted data from three waves: 2009, 2011, and 2015 (Supplementary Figure S1). Firstly, we included individuals in 2009 who were prediabetic, an asymptomatic status which was defined as the FPG level of 5.6–6.9 mmol/L and/or the HbA1c level of 5.7–6.4% with self-reported free from diabetes. Within 3533 individuals with prediabetes identified in 2009, 2328 followed up for three waves and had the self-report status of diabetes in 2015. Considering the robustness of GMM and the reliability of data, only individuals with complete data of BMI and MUAC in all three waves were enrolled. After eliminated missing values or outliers of target anthropometrics measurements (height, weight, and MUAC), 1529 participants of prediabetes were involved in order to assess the associations between BMI and MUAC trajectories and diabetes. A total of 56 cases (3.66%) of diabetes were observed, with means of follow-up time at 5.95 ± 0.45 years.

### 3.1. GMM for BMI and Upper Arm Circumference

Table S1 summarizes the fitness indices for targeted anthropometrics measurements trajectories (BMI and MUAC, respectively). 

For trajectories of BMI models, the *p*-value of aLMR-test showed a significant difference under the three-class model (*p* = 0.0115) which indicated that the three-class model was better than the two-class model, with the highest entropy value (0.954). However, BLRT indicated that the four-class model was the best one (*p* < 0.001), with slightly lower entropy (0.826). In order to solve this contradiction, we used a scree plot to determine the best model (Figure S2). The aBIC of BMI trajectories decreased sharply from 2-class to 4-class and rebounded from 4-class to 5-class. Thus, the best model for BMI trajectory was the four-class model.

For trajectories of MUAC models, the *p*-value of aLMR-test and BLRT both showed a significant difference in to the 4-class model (all *p* < 0.05), with an acceptable value of entropy (0.731). The scree plot of aBIC for MUAC trajectories followed the similar pattern with the one of BMI. Thus, the best fitting model for trajectories of MUAC was the 4-class model.

**Figure 1:**
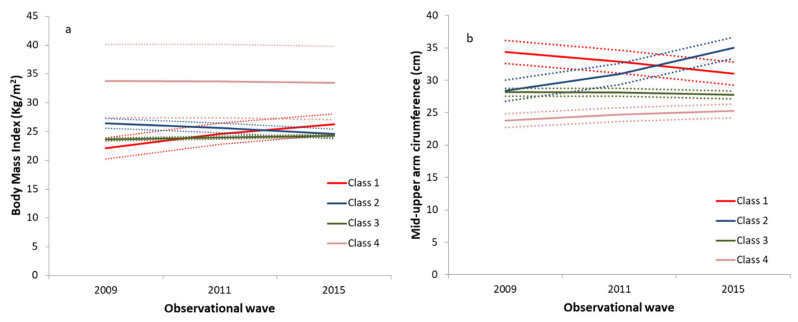
The BMI and MUAC trajectories modeled by GMM. (**a**) The plots for classifications of BMI trajectories by GMM. (**b**) The plots for classifications of MUAC trajectories by GMM. Abbreviations: BMI, body mass index; MUAC, mid-upper arm circumference; GMM, growth mixture model.

Figure 1 shows the best-fitting GMM models for BMI and MUAC trajectories. In the GMM model for BMI (Figure 1a), the majority of participants (green line, n = 1396) observed a low-stable BMI trajectory that began around 23.7 kg/m^2^. In addition, 6.9% of participants (blue line, n = 106) observed a middle-decline trajectory, and 0.9% of participants (red line, n = 14) observed a low-increase BMI trajectory. The rest of the participants observed a high-stable BMI trajectory above 33.0 kg/m^2^ (purple line, n = 13). For MUAC (Figure 1b), four trajectories were also observed: 3% for the high-decrease trajectory (red line, n = 46), 0.06% for the middle-increase trajectory (blue line, n = 9), 82.3% for the middle-stable trajectory (green line, n = 1259), and 14.1% for the low-increase trajectory (purple line, n = 215).

### 3.2. Characteristics across Trajectory Groups

Tables S2 and S3 show the characteristic of BMI and MUAC trajectory groups, respectively. Significant differences were observed between different location, gender, TSF, and activity level across trajectory groups for BMI (all *p* < 0.05). By contrast, more statistically significant differences were observed across trajectory groups for MUAC, including age, location, gender, energy intake, carbohydrate intake, TSF at baseline, and activity level at baseline (all *p* < 0.05). 

### 3.3. Logistic Regression and Receiver Operating Characteristic Curve for Diabetes

Table 1 and Table 2 represent the association between the trajectories of BMI and MUAC and diabetes, respectively (Models 1 to 4). As shown in Table 1, compared to individuals with prediabetes of low-stable BMI trajectory (the green line in Figure 1a), the ones with middle-decline (the blue line in Figure 1a) and high-stable BMI (the purple line in Figure 1a) were more likely to develop diabetes in the future after adjusted for various potential confounders (range of ORs: 3.309–4.219 and 7.103–10.060, respectively, model 2 to model 4). After being adjusted for potential confounders (Table 2), individuals with prediabetes of the high-decrease trajectory of MUAC (the red line in Figure 1b) were at a higher risk for developing diabetes compared to the ones with the middle-stable trajectory of MUAC (OR (95% CI): 3.085 (1.139–8.835), the green line in Figure 1b, model 4). Model five in Table 1 showed the relationship between diabetes and trajectory of BMI and MUAC after being adjusted for trajectories of MUAC and other multiple covariates when compared to the Class three; the participants in class two of the BMI trajectory reported significant positive association with diabetes (OR (95% CI): 3.139 (1.538–6.408), the blue line compared to the green line in Figure 1a).

**Figure 2:**
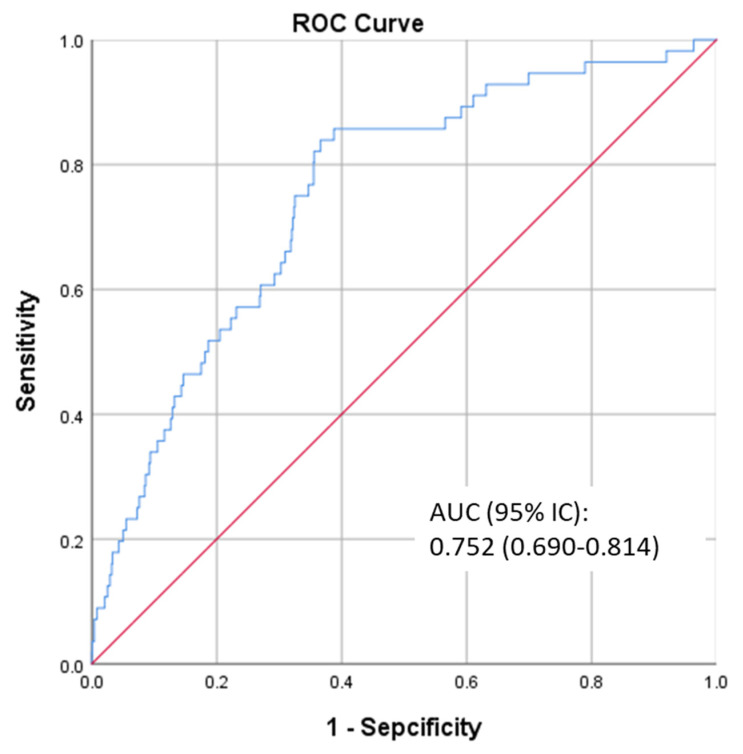
The ROC of the prediction model. Abbreviations: ROC, receiver operating characteristic curve; AUC, area under the curve.

Figure 2 show the receiver operating characteristic curve (ROC) of the prediction model for diabetes by the trajectories of BMI and MUAC (Model 5 in Table 1). The area under the curve (AUC) was 0.752 (95% CI: 0.690–0.814).

A simple cross-validation was applied by using logistic regression to explore the efficiency of the predicted model (model five in Table 1). The training set (n_1_ = 1070) reported that the AUC with 95%CI was 0.748 (0.671–0.826). The best cut-off was 0.044, the corresponding sensitivity was 0.698, and the corresponding specificity was 0.725 (Supplementary Figure S3). Using this cut-off, the validating set (n_2_ = 459) reported that sensitivity was 0.667, and specificity was 0.690.

Repeated analyses were conducted to assess the influence of missing values. Sensitivity analyses showed that patterns of risk between diabetes and BMI and MUCA trajectories were similar to the main analysis (Tables S4 and S5).

## 4. Discussion

Although previous studies have confirmed the association between BMI and diabetes, the association between the trajectories of anthropometric variables and diabetes among Chinese with prediabetes has not been elucidated. To the best of our knowledge, this is the first study based on a national cohort that explored the trajectories of BMI and MUAC among the Chinese general population with prediabetes and evaluated the associations between these trajectories and the risk of diabetes. Our study found that the trajectories of BMI and MUAC were categorized into four classes by GMM, respectively. Significant positive associations were detected for the certain trajectories of BMI and MUAC and the risk of diabetes. In addition, it was promising to utilize the trajectories of BMI and MUAC to predict diabetes among the Chinese with prediabetes. These findings could help provide new insight into the prevention and management of diabetes in China. 

Our study showed that individuals with prediabetes of a high level of BMI trajectory were more likely to develop diabetes in the future (range of ORs: 3.309–4.219 and 7.103–10.060, respectively; all *p* < 0.05). This was higher yet comparable with previous studies that discussed the association between BMI trajectories and risk of diabetes among the Chinese general population [15,17]. Mi, B. and colleagues studied 14,185 participants with the mean of 11.2 years of follow-up from the CHNS cohort and indicated that the substantial gain pattern was associated with a higher hazard of type 2 diabetes when compared with the stable pattern (adjusted hazard ratio [HR]: 1.49, 95%CI: 1.09–2.03) [15]. Another study, which was also based on the CHNS database, reported similar results: Compared with the low-increasing group, adjusted HR and 95% CIs were 1.21 (0.99 to 1.48) and 1.56 (1.06 to 2.30) for the medium-increasing and high-increasing group, respectively [17]. Compared to these two studies conducted among the general population, the higher risk reported in our study was understandable because prediabetes was a risk factor for diabetes [5]; thus, the study conducted among individuals with prediabetes reported higher risk. However, evidence from studies conducted among individuals with prediabetes were scarce. Two previous studies have examined weight change in the progression from prediabetes to diabetes, although their findings were not directly comparable to our study due to the difference in study design and data analysis [18,42]. Hu, H. and colleagues conducted a cohort study enrolling 22,945 individuals with prediabetes among Japanese people [18]. They found out that people who progressed to diabetes had a larger increase in mean BMI from 7 years to 1 year before diagnosis, which was about three times that of people who remained with prediabetes [18]. By contrast, a smaller study conducted in Singapore (n = 297) indicated that people who developed diabetes appeared to have a smaller weight gain than those who remained with prediabetes [42]. 

We found that individuals with prediabetes with a high-decrease pattern of MUAC trajectory were at a statistically significant high risk of diabetes, even after being adjusted for the TSF and other confounders. Although no previous similar studies were identified, several indirect evidence supported our hypothesis that the association may relate to the abnormal glucose metabolism and loss of skeleton muscles in the progression to diabetes. The MUAC was a measurement of the sum of the muscle and subcutaneous fat in the upper arm [19]. Previous studies pointed out that large MUAC was associated with insulin resistance [20,21]. In addition, the decrease in MUAC may indicate the loss of skeletal muscles [19]; the skeletal muscle was one of the most relevant components of glucose metabolic capacity of diabetes [43]. Under euglycemic hyperinsulinemic conditions, the skeletal muscle undertook 80% of glucose uptake [44]. The loss of skeleton muscle may impair the capacity of glucose metabolic and result in an increased risk of diabetes. As to individuals with a high-decrease pattern of MUAC trajectory in our study, the high MUAC indicated that they were insulin resistant at the beginning and the decreasing trend implicated that their glucose metabolic capacity gradually deteriorated during the observational period; thus, they were more likely to develop diabetes. 

The distinct trajectories in our study suggest that monitoring BMI and/or MUAC over time can help identify those at a higher risk of diabetes. In addition, the AUC values of logistic regression models implied the feasibility of trajectories of BMI and MUAC to predict diabetes among the high-risk population. Predicting individuals’ future risk of diabetes enables medical personnel to provide tailored programs of prevention and management. For example, for individuals classified in the high BMI and/or high trajectory of MUAC, more intensive monitoring and intervention may be required to delay or even prevent its progression to diabetes. The American Diabetes Association (ADA), the European Society of Cardiology (ESC), and the Chinese Diabetes Society (CDS) recommended identifying and intervening prediabetes in the early stage [45,46,47]. The three academies all recommended lifestyle changes for prediabetes, such as diet, physical activity, and bodyweight reduction; however, subtle differences existed [45,46,47]. With respect to physical activity recommendations, both ADA and ESC pointed out the importance of resistance training for improving insulin sensitivity and specifically recommended resistance training [45,46]. By contrast, in the guideline of CDS, no resistance training was mentioned for prediabetes [47]. In addition to improving insulin sensitivity, resistance training has been thought to promote increases in muscle strength and mass [48,49]. Previous studies have shown that Asians have the lower fat-free mass index and a higher prevalence of diabetes compared to Westerners [50,51]. Thus, it is particularly important for Chinese medical faculties to realize the significance of resistance training for Chinese people in order to prevent diabetes. A revision regarding resistance training to maintain muscle mass for prediabetes was required for the CDS guideline in the new version.

With a large sample size from a national cohort, the statistical power and representativeness of this study were warranted. By applying GMM, we were able to accurately categorize subgroup trajectories of BMI and MUAC, which followed similar growth patterns. However, our study had several limitations. Firstly, due to the limited biochemical information, the prediabetes in this study only included IFG and individuals with a moderate level of HbA1c (5.7–6.4%); the IGT individuals were left out. Thus, it is worth noting when comparing this study to others. Secondly, it was a pity that we gave up analyzing the association between the risk of diabetes and the arm muscle area (AMA), which was a more direct indicator of skeletal muscle mass than MUAC, due to the concern of potential large observation error in AMA measurements [52]. For amendment, we adjusted TSF in the logistic regression model for MUAC trajectories and observed that certain MUAC trajectories were an independent risk factor for diabetes among individuals with prediabetes. Thirdly, the status of diabetes and several other covariates were self-reported; thus, potential measurement bias may exist, such as misclassification of status of diseases from interviewers, recalling bias, and self-report bias for interviewees. Fourthly, missing values may bias the result. Nonetheless, the effect of missing values might be minimized because the proportion was rather low (less than 5%) and the association between BMI and MUAC trajectories and diabetes remained unchanged without imputation of missing values. Fifthly, the temporality between exposures and the outcome may be blurred in this study. Sixth, some other covariates, such as genes, family history, and diet patterns, were not evaluated as potential confounding factors due to the limitation of the original raw data. In order to obtain the causal relationship, studies with larger sample size and more strict design were required. 

## 5. Conclusions

The current study grouped distinct trajectories of BMI and MUAC for individuals with prediabetes. A significant association between different trajectory groups and the risk of diabetes was observed. High-stable BMI and high-decrease MUAC pattern were independent risk factors for diabetes. Diabetes prevention programs should emphasize the significance of maintaining skeletal muscle mass, and resistance training should be recommended for prediabetes. 

## Figures and Tables

**Table 1:** The associations between BMI trajectories and diabetes by logistic regressions.

Models	Variable	OR (95% CI)	*p*
Model 1	Class 3 BMI	Ref	-
	Class 2 BMI	4.219 (2.145–8.298)	0.000
	Class 1 BMI	-	-
	Class 4 BMI	9.915 (2.630–37.379)	0.001
Model 2	Class 3 BMI	Ref	-
	Class 2 BMI	3.924 (1.959–7.861)	0.000
	Class 1 BMI	-	-
	Class 4 BMI	10.050 (2.582–39.119)	0.001
Model 3	Class 3 BMI	Ref	-
	Class 2 BMI	3.634 (1.795–7.356)	0.000
	Class 1 BMI	-	0.999
	Class 4 BMI	10.060 (2.510–40.316)	0.001
Model 4	Class 3 BMI	Ref	-
	Class 2 BMI	3.309 (1.626–6.735)	0.001
	Class 1 BMI	-	-
	Class 4 BMI	7.103 (1.673–30.147)	0.008
Model 5	Class 3 BMI	Ref	-
	Class 2 BMI	3.139 (1.538–6.408)	0.002
	Class 1 BMI	-	-
	Class 4 BMI	4.639 (0.967–22.259)	0.055
	Class 3 MUAC	Ref	-
	Class 2 MUAC	-	-
	Class 1 MUAC	2.181 (0.733–6.491)	0.161
	Class 4 MUAC	0.579 (0.198–1.689)	0.317

Abbreviations: BMI, body mass index; OR, odd ratio; CI, confidence interval; MUAC, mid-upper arm circumference. Model 1: classes of trajectories of BMI only; Model 2: additionally adjusted for education level, location, ethnicity, gender, and age at 2009 based on model 1; Model 3: additionally adjusted for smoking at 2009, drinking at 2009, drink tea at 2009, the average of 3 days energy intake at 2009, the average of 3 days carbohydrate intake at 2009, and activity level at 2009 based on model 2; Model 4: additionally adjusted for TSF based on model 3; Model 5: additionally adjusted for classes of trajectories of MUAC based on model 4.

**Table 2:** The associations between MUAC trajectories and diabetes by logistic regression.

Models	Variable	OR (95% CI)	*p*
Model 1	Class 3 MUAC	Ref	-
	Class 2 MUAC	-	-
	Class 1 MUAC	3.955 (1.597–9.799)	0.003
	Class 4 MUAC	0.500 (0.178–1.403)	0.188
Model 2	Class 3 MUAC	Ref	-
	Class 2 MUAC	-	-
	Class 1 MUAC	4.336 (1.718–10.944)	0.002
	Class 4 MUAC	0.466 (0.164–1.326)	0.152
Model 3	Class 3 MUAC	Ref	-
	Class 2 MUAC	-	-
	Class 1 MUAC	4.127 (1.614–10.557)	0.003
	Class 4 MUAC	0.467 (0.164–1.333)	0.155
Model 4	Class 3 MUAC	Ref	-
	Class 2 MUAC	-	-
	Class 1 MUAC	3.085 (1.139–8.356)	0.027
	Class 4 MUAC	0.554 (0.191–1.608)	0.278

Abbreviations: MUAC, mid-upper arm circumference; OR, odd ratio; CI, confidence interval. Model 1: classes of trajectories of MUAC only; Model 2: additionally adjusted for education level, location, ethnicity, gender, and age at 2009 based on model 1; Model 3 additionally adjusted for smoking at 2009, drinking at 2009, drink tea at 2009, the average of 3 days energy intake at 2009, the average of 3 days carbohydrate intake at 2009, and activity level at 2009 based on model 2; Model 4 additionally adjusted for TSF based on model 3.

## Data Availability

The data that support the findings of this study are available from the China Health and Nutrition Survey (CHNS) but restrictions apply to the availability of these data, which were used under license for the current study and so are not publicly available. Data are, however, available from the authors upon reasonable request and with permission of CHNS.

## Supplementary Materials

The following are available online at https://www.mdpi.com/article/10.3390/nu13124356/s1, Table S1: Fitness indices for two-class to five-class growth mixture models for body mass index and mid-upper arm circumference among Chinese pre-diabetes individuals, Table S2: The characteristics across body mass index trajectory groups (N = 1529), Table S3: The characteristics across mid-upper arm circumference trajectory groups (N = 1529), Table S4: The associations between BMI trajectories and diabetes by logistic regressions without missing values, Table S5: The associations between MUAC trajectories and diabetes by logistic regressions without missing values, Figure S1: The flow chart of enrollment, Figure S2: The scree plots of GMM, Figure S3: The receiver operator characteristic curve of training set (n_1_ = 1070).
