# Supplementary material for: The Association between Trajectories of Anthropometric Variables and Risk of Diabetes among Prediabetic Chinese
Source: Nutrients. 2021 Dec 3;13(12):4356. doi: 10.3390/nu13124356 (PMC8706558; doi:10.3390/nu13124356)
Supplement: Supplementary file 1 [file nutrients-13-04356-s001.zip › nutrients-1489076-supplementary.pdf]

Table S1. Fitness indices for two-class to five-class growth mixture models for body mass index and mid-upper arm circumference among Chinese pre-diabetes individuals.

Outcomes	Classes	K	G²/LL	aBIC	Entropy	aLMR	BLRT	Class proportions	Class counts
BMI	2	11	-9195.397	18436.505	0.948	0.0208	<0.001	0.00981/0.99019	15/1514
	3	14	-9187.991	18434.161	0.954	0.0115	0.005	0.01177/0.00458/0.98365	18/7/1504
	**4**	**17**	**-9174.365**	**18419.375**	**0.826**	**0.065**	**<0.001**	**0.00916/0.06933/0.91302/0.0085**	**14/106/1396/13**
	5	20	-9170.413	18423.939	0.638	0.5224	0.155	0.08371/0.00785/0.01112/0.74689/0.15043	128/12/17/1142/230
MUAC	2	11	-10759.064	21563.84	0.939	0.1002	0.1091	0.99411/0.00589	1520/9
	3	14	-10752.7	21563.58	0.767	0.4771	0.4894	0.93525/0.02485/0.0399	1430/38/61
	**4**	**17**	**-10745.685**	**21562.016**	**0.731**	**0.0431**	**0.0470**	**0.03009/0.00589/0.82341/0.14061**	**46/9/1259/215**
	5	20	-10739.785	21562.683	0.700	0.2478	0.2609	0.00981/0.03074/0.09941/0.81818/0.04186	15/47/152/1251/64

Abbreviation: BMI, Body mass index; MUAC, mid-upper arm circumference; G²/LL, likelihood ratio chi-square; aBIC, sample size-adjusted Bayesian Information Criterion; aLMR, adjusted Lo–Mendell–Rubin likelihood ratio test; BLRT, bootstrapped likelihood ratio test.

Table S2. The characteristics across body mass index trajectory groups (N = 1529).

Variable	Class 3 (n = 1396)		Class 2 (n = 106)		Class 1(n = 14)		Class 4(n = 13)		*p*
	N	%	N	%	N	%	N	%	
**Cases**	41	2.9%	12	11.3%	0	0.0%	3	23.1%	<0.001[b]
**Age** [a]	54.4 ±11.8		54.8 ±12.2		59.6 ±12.8		54.1 ±8.4		0.434
**Location**									
urban	335	24.0%	39	36.8%	6	42.9%	3	23.1%	0.012[b]
rural	1061	76.0%	67	63.2%	8	57.1%	10	76.9%	
**Ethnic**									
Majority (Han)	1288	92.3%	93	87.7%	11	78.6%	13	100.0%	0.075[b]
Minority	108	7.7%	13	12.3%	3	21.4%	0	0.0%	
**Gender**									
Male	623	44.6%	35	33.0%	5	35.7%	3	23.1%	0.044
Female	773	55.4%	71	67.0%	9	64.3%	10	76.9%	
**Education level**									
Illiteracy	399	28.6%	27	25.5%	7	50.0%	3	23.1%	0.873[b]
Primary school	305	21.8%	25	23.6%	3	21.4%	4	30.8%	
Lower middle school degree	425	30.4%	31	29.2%	2	14.3%	4	30.8%	
Upper middle school degree	134	9.6%	13	12.3%	1	7.1%	2	15.4%	
Technical or vocational degree and higher	133	9.5%	10	9.4%	1	7.1%	0	0.0%	
**Smoking at baseline**									
Never	957	68.6%	84	79.2%	11	78.6%	11	84.6%	0.066[b]
Ever	439	31.4%	22	20.8%	3	21.4%	2	15.4%	
**Drinking at baseline**									
Never	930	66.6%	75	70.8%	9	64.3%	8	61.5%	0.0786[b]
Ever	466	33.4%	31	29.2%	5	35.7%	5	38.5%	

**Drinking tea at baseline**									
Never	911	65.3%	65	61.3%	9	64.3%	8	61.5%	0.848[b]
Ever	485	34.7%	41	38.7%	5	35.7%	5	38.5%	
**Energy intake at baseline(kcal)** [b]	2170.9 ±656.0		2153.3 ±806.3		2271.8 ±728.5		2147.5 ±730.1		0.939
**Carbohydrate intake at baseline(g)** [b]	301.2 ±99.8		301.2 ±99.8		284.1 ±106.0		349.0 ±125.5		0.108
**Triceps skin fold at baseline (mm)** [a]	16.8 ± 8.0		20.3 ± 8.4		14.0 ± 8.0		28.5 ± 9.7		<0.001
**Activity level at baseline**									
Light	669	47.9%	76	71.7%	6	42.9%	6	46.2%	<0.001[b]
Middle	163	11.7%	7	6.6%	1	7.1%	2	15.4%	
Heavy	559	40.0%	22	20.8%	7	50.0%	4	30.8%	
No working ability	5	0.4%	1	0.9%	0	0.0%	1	7.7%	

[a] Presented by mean ± standard deviation; [b] Tested by Fisher's exact tests.

Table S3. The characteristics across mid-upper arm circumference trajectory groups (N = 1529).

Variable	Class 3 (n = 1259)		Class 2 (n = 9)		Class 1(n = 46)		Class 4(n = 215)		*p*
	N	%	N	%	N	%	N	%	
**Cases**	46	3.7%	0	0.0%	6	13.0%	4	1.9%	<0.001[c]
**Age** [a]	54.0(16.0)		57.0(28.5)		49.5(13.3)		61(21)		<0.001[d]
**Location**									
urban	315	25.0%	2	22.2%	11	23.9%	55	25.6%	0.012[b]
rural	944	75.0%	7	77.8%	35	76.1%	160	74.4%	
**Ethnic**									
Majority (Han)	1169	92.9%	9	100.0%	44	95.7%	183	85.1%	0.075
Minority	90	7.1%	0	0.0%	2	4.3%	32	14.9%	
**Gender**									
Male	566	45.0%	6	66.7%	18	39.1%	76	35.3%	0.044
Female	693	55.0%	3	33.3%	28	60.9%	139	64.7%	
**Education level**									
Illiteracy	336	26.7%	2	22.2%	12	26.1%	86	40.0%	0.873
Primary school	281	22.3%	2	22.2%	9	19.6%	45	20.9%	
Lower middle school degree	401	31.9%	2	22.2%	17	37.0%	42	19.5%	
Upper middle school degree	124	9.8%	2	22.2%	6	13.0%	18	8.4%	
Technical or vocational degree and higher	117	9.3%	1	11.1%	2	4.3%	24	11.2%	
**Smoking at baseline**									
Never	874	69.4%	5	55.6%	34	73.9%	150	69.8%	0.066
Ever	385	30.6%	4	44.4%	12	26.1%	65	30.2%	
**Drinking at baseline**									
Never	831	66.0%	5	55.6%	27	58.7%	159	74.0%	0.786[c]
Ever	428	34.0%	4	44.4%	19	41.3%	56	26.0%	
**Drinking tea at baseline**									
Never	817	64.9%	8	88.9%	31	67.4%	137	63.7%	0.848[c]
Ever	442	35.1%	1	11.1%	15	32.6%	78	36.3%	
**Energy intake at baseline(kcal)** [b]	2198.6 ±673.0		2288.1 ±408.5		1984.3 ±516.6		2040.3 ±658.3		0.0030
**Carbohydrate intake at baseline(g)** [b]	303.4 ±101.5		324.7 ±79.6		272.0 ±93.1		287.8 ±98.3		0.0340
**Triceps skin fold at baseline (mm)** [a]	17.0(10.7)		20.0(18.3)		24.8(12.7)		10.7(8.7)		<0.001[d]
**Activity level at baseline**									
Light	605	48.1%	6	66.7%	27	58.7%	119	55.3%	<0.001[b]
Middle	152	12.1%	0	0.0%	6	13.0%	15	7.0%	
Heavy	496	39.4%	3	33.3%	12	26.1%	81	37.7%	
No working ability	6	0.5%	0	0.0%	1	2.2%	0	0.0%	

[a] Presented by median (interquartile range); [b] Presented by mean ± standard deviation; [c] Tested by Fisher's exact tests; [d] Tested by Kruskal–Wallis tests.

Table S4. The associations between BMI trajectories and diabetes by logistic regressions without missing values.

Models	Variable	OR (95% CI)	*p*
Model 1	Class 3 BMI	Ref	-
	Class 2 BMI	4.219 (2.145-8.298)	0.000
	Class 1 BMI	-	-
	Class 4 BMI	9.915 (2.630-37.379)	0.001
Model 2	Class 3 BMI	Ref	-
	Class 2 BMI	4.018 (2.001-8.065)	0.000
	Class 1 BMI	-	-
	Class 4 BMI	10.278 (2.642-39.984)	0.001
Model 3	Class 3 BMI	Ref	-
	Class 2 BMI	3.787 (1.864-7.693)	0.000
	Class 1 BMI	-	-
	Class 4 BMI	10.092 (2.478-41.097)	0.001
Model 4	Class 3 BMI	Ref	-
	Class 2 BMI	3.478 (1.699-7.118)	0.001
	Class 1 BMI	-	-
	Class 4 BMI	7.089 (1.644-30.571)	0.009
Model 5	Class 3 BMI	Ref	-
	Class 2 BMI	3.290 (1.602-6.756)	0.001
	Class 1 BMI	-	-
	Class 4 BMI	4.590 (0.949-22.215)	0.058
	Class 3 MUAC	Ref	-
	Class 2 MUAC	-	-
	Class 1 MUAC	2.248 (0.757-6.676)	0.145
	Class 4 MUAC	0.606 (0.207-1.773)	0.361

Abbreviations: BMI, body mass index; OR, odd ratio; CI, confidence interval; MUAC, mid-upper arm circumference. Model 1: classes of trajectories of BMI only; Model 2: additionally adjusted for education level, location, ethnicity, gender, and age at 2009 based on model 1; Model 3: additionally adjust for smoking at 2009, drinking at 2009, drink tea at 2009, the average of 3 days energy intake at 2009, the average of 3 days carbohydrate intake at 2009, and activity level at 2009 based on model 2; Model 4: additional adjustment for triceps skin fold based on model 3; Model 5: additional adjustment for classes of trajectories of MUAC based on model 4.

Table S5. The associations between MUAC trajectories and diabetes by logistic regressions without missing values.

Models	Variable	OR (95% CI)	*p*
Model 1	Class 3 MUAC	Ref	-
	Class 2 MUAC	-	-
	Class 1 MUAC	3.955 (1.597-9.799)	0.003
	Class 4 MUAC	0.500 (0.178-1.403)	0.188
Model 2	Class 3 MUAC	Ref	-
	Class 2 MUAC	-	-
	Class 1 MUAC	4.405 (1.745-11.121)	0.002
	Class 4 MUAC	0.473 (0.166-1.347)	0.161
Model 3	Class 3 MUAC	Ref	-
	Class 2 MUAC	-	-
	Class 1 MUAC	4.271 (1.659-10.994)	0.003
	Class 4 MUAC	0.482 (0.169-1.378)	0.173
Model 4	Class 3 MUAC	Ref	-
	Class 2 MUAC	-	-
	Class 1 MUAC	3.191 (1.178-8.645)	0.022

Class 4 MUAC	0.579 (0.199-1.681)	0.315

Abbreviations: MUAC, mid-upper arm circumference; OR, odd ratio; CI, confidence interval.
Model 1: classes of trajectories of MUAC only; Model 2: additionally adjusted for education level, location, ethnicity, gender, and age at 2009 based on model 1; Model 3 additionally adjusted for smoking at 2009, drinking at 2009, drink tea at 2009, the average of 3 days energy intake at 2009, the average of 3 days carbohydrate intake at 2009, and activity level at 2009 based on model 2; Model 4 additionally adjusted for triceps skinfold (TSF) based on model 3.

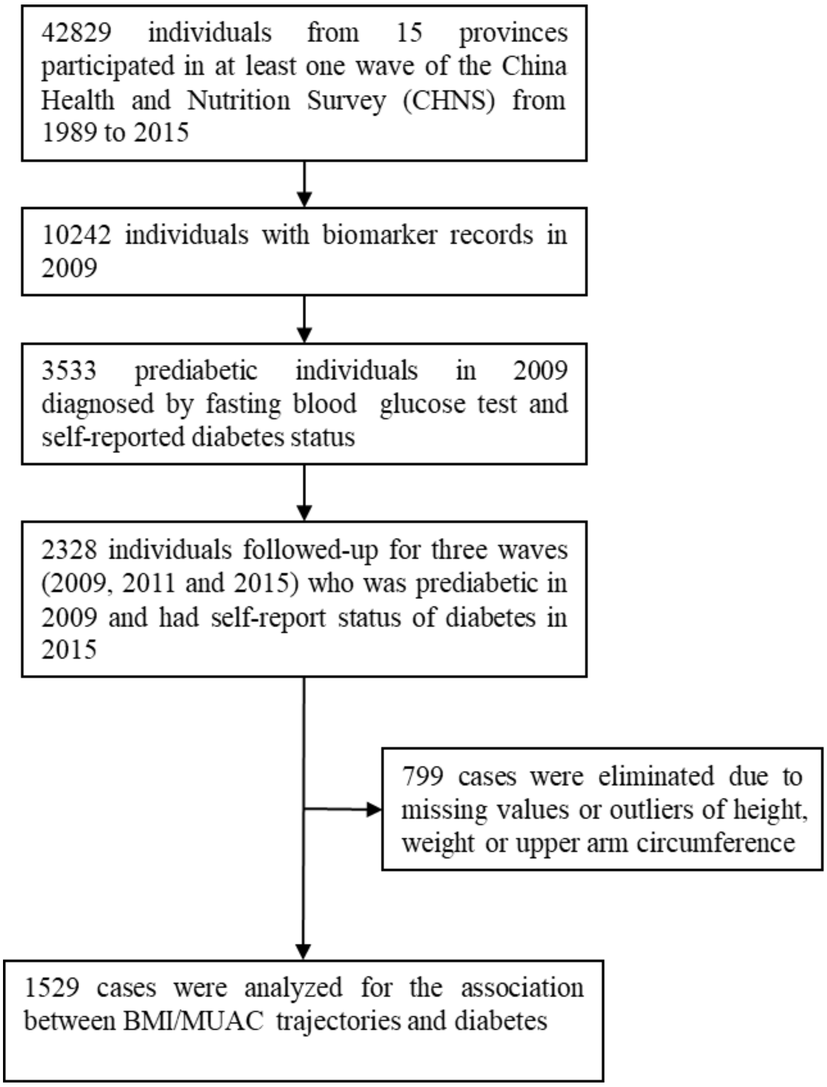

Figure S1. The flow chart of enrollment

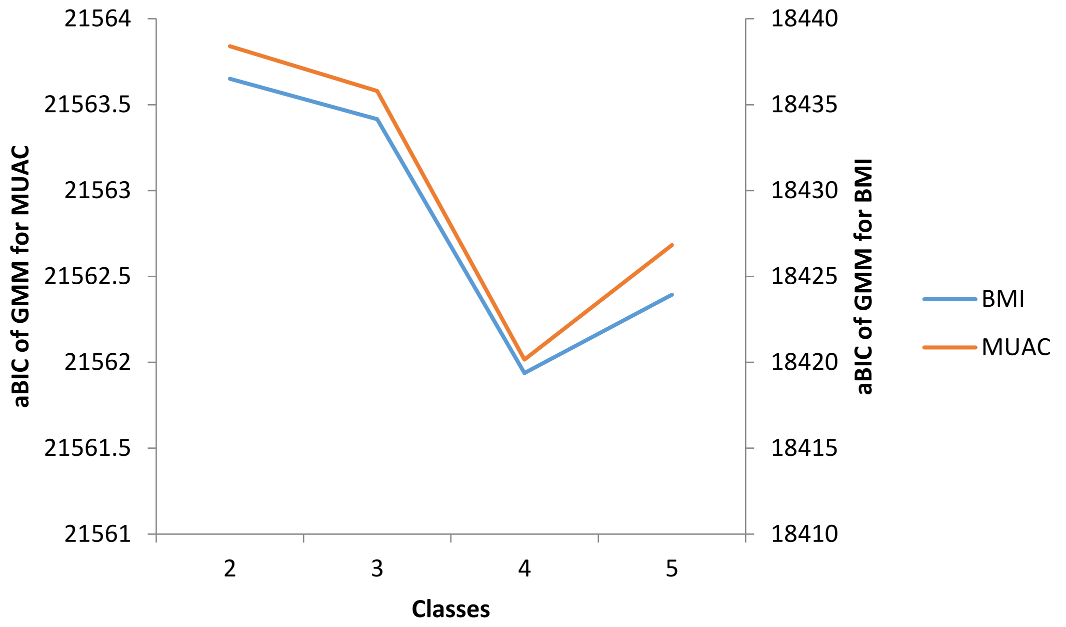

Figure S2. The scree plots of GMM. Abbreviations: GMM, growth mixture models; BMI, body mass index; MUAC, mid-upper arm circumference.

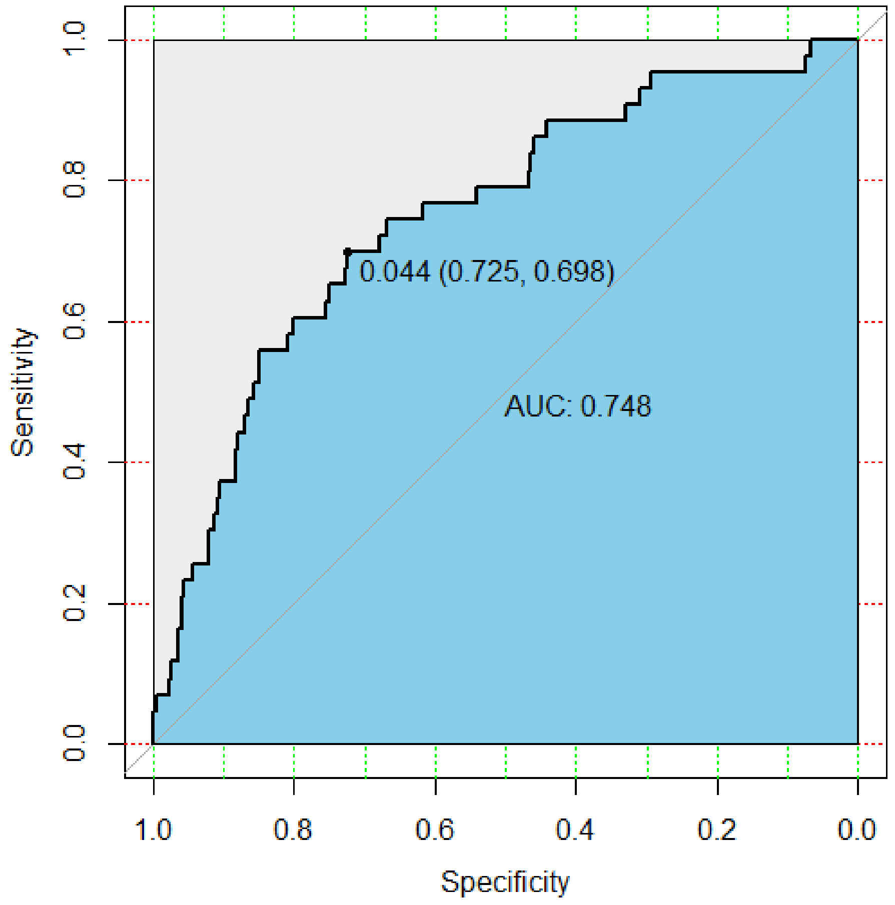

Figure S3. The receiver operator characteristic curve of training set (n1 = 1070). Abbreviations: AUC, area under the curve. The AUC with 95%CI was 0.748 (0.671-0.826). The best cut-off was 0.044, the corresponding sensitivity was 0.698, and the corresponding specificity was 0.725.
